# Acknowledgement of manuscript reviewers 2014

**DOI:** 10.1186/1742-4755-12-8

**Published:** 2015-03-23

**Authors:** José M Belizán, Sunni Mumford

**Affiliations:** Institute for Clinical Effectiveness and Health Policy, Buenos Aires, Argentina; Division of Intramural Population Health Research, of the Eunice Kennedy Shriver National Institute of Child Health and Human Development, National Institutes of Health, Rockville, Maryland

## Abstract

The editors of *Reproductive Health* would like to extend a sincere thank you to all our valued reviewers listed below who contributed to the journal in Volume 11 (2014). The journal has grown 63% in submissions and 43% in publications compared to 2013 and therefore the co-operation and dedicated time of peer reviewers is increasingly essential in order to move these manuscripts through the submission system so high quality research does not remain static and unpublished. We are very pleased to have a diverse range of reviewers from high, middle and low income countries, contributing to the dissemination of research findings in an open access journal. Please accept our sincerest and deepest thanks for your knowledge, time and continuing efforts.

**Aniekan Abasiattai**

Nigeria

**Shelly Natasha Abdool**

Panama

**Sileshi Garoma Abeya**

Ethiopia

**Suniti Acharya**

Nepal

**Ishag Adam**

Sudan

**Susan Adamchak**

United States of America

**Richard Adanu**

Ghana

**Ramesh Adhikari**

Nepal

**Sohail Agha**

United States of America

**Tahera Ahmed**

Bangladesh

**Adetoun Akinwusi**

Nigeria

**Nazmul Alam**

Canada

**Hesham Al-Inany**

Egypt

**Avni Amin**

Switzerland

**Irene Andia-Biraro**

Uganda

**Augustine Ankomah**

Ghana

**Bao-Yi Ann**

Taiwan

**Mudassir Anwar**

Malaysia

**Laura Arbour**

Canada

**Antenah Asefa**

Ethiopia

**Hussen Mekonnen Asfaw**

Ethiopia

**Ian Askew**

Kenya

**Shervin Assari**

United States of America

**Berhanemeskel Atsbeha**

Ethiopia

**José Ayres**

Brazil

**Stella Babalola**

United States of America

**Rebecca Bailey**

United States of America

**Robert Bailey**

United States of America

**Ariel Bardach**

Argentina

**Alka Barua**

India

**Angela M Bayer**

United States of America

**Fatemeh Bazarganipour**

Iran

**Rachel Beanland**

United Kingdom

**Mrutyunjaya Bellad**

India

**Regina Benevides**

United States of America

**Peter Bjerregaard**

Denmark

**Alemayehu Bogale**

Ethiopia

**Stephan Brenner**

Germany

**Iracema MP Calderon**

Brazil

**Christopher Carroll**

United Kingdom

**Janet Catov**

United States of America

**Francesca Cavallaro**

United Kingdom

**Venkatraman Chandra-Mouli**

Switzerland

**Collins Chansa**

Zambia

**Subidita Chaterjee**

India

**Dafang Chen**

China

**Amsale Cherie**

Ethiopia

**Z. Mike Chirenje**

Zimbabwe

**Tim Colbourn**

United Kingdom

**Selby Conrad**

United States of America

**Deborah Constant**

South Africa

**Diane Cooper**

South Africa

**Divanise Correia**

Brazil

**Vanessa Dalton**

United States of America

**Eugene Kofuor Maafo Darteh**

Ghana

**Anindita Dasgupta**

United States of America

**Daniel Datiko**

Ethiopia

**Nancy Day**

United States of America

**Caroline de Costa**

Australia

**Korrie de Koning**

Netherlands

**Gurmesa Tura Debelew**

Ethiopia

**Peter Decat**

Belgium

**Kebede Deribe**

Ethiopia

**Claudia Diaz Olavarrieta**

Mexico

**Nagasa Dida**

Ethiopia

**Martha Dirnfeld**

Israel

**Mai Do**

United States of America

**Amol Dongre**

India

**Melba D'Souza**

Oman

**Putu Duff**

Canada

**Jill Durocher**

United States of America

**Shahul Ebrahim**

United States of America

**Anke Ehrhardt**

United States of America

**Omaima El-Gibaly**

Egypt

**Cherrie Lynn Evans**

United States of America

**Alexandre Faisal-Cury**

Brazil

**Cristina Faludi**

Romania

**Wael Fayek**

Egypt

**Xing Lin Feng**

China

**Sonia Fernandez-Balbuena**

Spain

**Tamara Fetters**

United States of America

**Sarah Finocchario Kessler**

United States of America

**Virginia Fonner**

United States of America

**Edward Fottrell**

United Kingdom

**Tine Gammeltoft**

Denmark

**Kaberi Gayen**

Bangladesh

**Abebaw Gebeyehu**

Ethiopia

**Samson Gebremedhin**

Ethiopia

**Kasiye Gemechu**

Ethiopia

**Lindsey Gottschalk**

United States of America

**Dana Greeson**

United States of America

**Sorina Grisaru-Granovsky**

Israel

**Nermin Gürhan**

Turkey

**Eshetu Gurmu**

Ethiopia

**Juan Pablo Gutierrez**

Mexico

**Randi Hagerman**

United States of America

**Demewoz Haile**

Ethiopia

**Tsegazeab Hailu**

Ethiopia

**Karin Hammarberg**

Australia

**Karin Hannes**

Belgium

**Jane Harries**

South Africa

**Emily Harville**

United States of America

**Lisa Hirschhorn**

United States of America

**Sarah Jane Holcombe**

United States of America

**Caroline Homer**

Australia

**Andrea Hoopes**

United States of America

**Sennen Hounton**

United States of America

**Justine Hsu**

Switzerland

**Lawrence Ikamari**

Kenya

**Chimaraoke Izugbara**

Nigeria

**Meenakshi Jain**

India

**Victoria Jennings**

United States of America

**Marianne Johansson**

Sweden

**Niels Jørgensen**

Denmark

**Allen Kabagenyi**

Uganda

**Humayun Kabir**

Bangladesh

**Fatch Welcome Kalembo**

China

**Gizat Molla Kassie**

Swaziland

**Leila Katz**

Brazil

**Dan Kaye**

Uganda

**Method Kazaura**

Tanzania

**Carl Kendall**

United States of America

**Amna Khan**

Pakistan

**Tahir Khan**

Malaysia

**Charles Kiggundu**

Uganda

**Mary Kinney**

South Africa

**Kerri Kissell**

United States of America

**Kim Klein**

Netherlands

**Marion Koso-Thomas**

United States of America

**Chen-I Kuan**

Taiwan

**Lucy Kululanga**

Malawi

**Kelly Kuo**

United States of America

**Betty Kwagala**

Uganda

**Amos Laar**

Ghana

**Malinee Laopaiboon**

Thailand

**Dennis Laryea**

Ghana

**Maria Leal**

Brazil

**Elena Lebetkin**

United States of America

**Jami Leichliter**

United States of America

**Kelly L'Engle**

United States of America

**Christine Leyns**

Belgium

**Wilson Liambila**

Kenya

**Yang Chu Lin**

Taiwan

**Nancy Liu**

United States of America

**Alejandra Lopez Gómez**

Uruguay

**Isabelle Lorraine**

United Kingdom

**Mona Loutfy**

Canada

**Kizito Lubano**

Kenya

**Fernando Luiz Affonso Fonseca**

Brazil

**Rebecka Lundgren**

United States of America

**Courtney Lynch**

United States of America

**Patricia Machawira**

South Africa

**Moke Magoma**

Tanzania

**Jennifer Malinowski**

United States of America

**Mats Malqvist**

Sweden

**Jacob R.S. Malungo**

Zambia

**Donald Marazzo**

United States of America

**Roberto Marci**

Italy

**Melisa Martínez Álvarez**

United Kingdom

**Wellington Martins**

Brazil

**Anthony Massinde**

Tanzania

**Rooyen Mavenyengwa**

Namibia

**Michael Mbizvo**

Switzerland

**Elizabeth McClure**

United States of America

**Sarah McDonald**

Canada

**Tamara Melnik**

Brazil

**Tesfaye Mengistu**

Ethiopia

**Philippe Merviel**

France

**Vildan Mevsim**

Turkey

**Kim Miller**

United States of America

**Samuel Mills**

United States of America

**Abdurahman Mohammed**

Ethiopia

**Lamiya Mohiyiddeen**

United Kingdom

**Ben Mol**

Afghanistan

**Allisyn Moran**

United States of America

**Mahmood Morshedi**

United States of America

**Eduardo Motta**

Brazil

**Cheryl Moyer**

United States of America

**Elias Mpofu**

Australia

**Projestine Muganyizi**

Tanzania

**Peter Mukasa**

Uganda

**Sunni Mumford**

United States of America

**Eunice Muthengi**

Kenya

**Sandra Nakic Rados**

Croatia

**Priya Nanda**

India

**Ahmed Nasr**

Egypt

**Mercy Nassali**

Bangladesh

**Ernest Ng**

Hong Kong

**Stella Maris Nicolau**

Brazil

**Dabere Nigatu**

Ethiopia

**Francis Obare**

Kenya

**Zeliha Öcek**

Turkey

**Jon Oyvind**

Norway

**Kanayo Ogujiuba**

Nigeria

**Ekechi Okereke**

Nigeria

**Pius Okong**

Uganda

**Samuel Olowookere**

Nigeria

**Alfred Osoti**

Kenya

**Easmon Otupiri**

Ghana

**Carter Owen**

United States of America

**Ozlem Ozdegirmenci**

Turkey

**Allison Pack**

United States of America

**Katalin Papp**

Hungary

**John Park**

United States of America

**Sheila Patel**

United States of America

**Mandira Paul**

Sweden

**Joana Pauleta**

Portugal

**Erin Pearson**

United States of America

**Brennan Peterson**

United States of America

**Karen Phillips**

Canada

**Carrie Purcell**

United Kingdom

**Anisur Rahman**

Bangladesh

**Salima Rahman**

Bangladesh

**Sunanda Ray**

Zimbabwe

**Krishna Regmi**

United Kingdom

**Olivia Remes**

Canada

**Jennifer Requejo**

United States of America

**Narjis Rizvi**

Pakistan

**Helen Rockliff**

United Kingdom

**Nicolette Roman**

South Africa

**Lori Ross**

Canada

**Sarah Saleem**

Pakistan

**A Sathiya Susuman**

South Africa

**Rebecca Schlaff**

United States of America

**Abdulbasit Seid**

Ethiopia

**Salaam Semaan**

United States of America

**Agumasie Semahegn**

Ethiopia

**Argentina Servin**

United States of America

**Bat Zion Shachar**

United States of America

**Iqbal Shah**

Switzerland

**Babar Tasneem Shaikh**

Pakistan

**Gerald Shambira**

Zimbabwe

**Ling Shi**

United States of America

**Oluwafolahan Sholeye**

Nigeria

**Binjwala Shrestha**

Nepal

**Estelle Monique Sidze**

Kenya

**Charalambos Siristatidis**

Greece

**Dragan Soldo**

Croatia

**Andrea Solnes Miltenburg**

Netherlands

**Ilene Speizer**

United States of America

**Joar Svanemyr**

Switzerland

**Monica Tarcea**

Romania

**Dessalegn Tesso**

Ethiopia

**Narbada Thapa**

Nepal

**Tizta Tilahun**

Ethiopia

**Farhan Tirmizi**

Pakistan

**Maria Regina Toloi**

Brazil

**Maria Regina Torloni**

Brazil

**Cheryl Vamos**

United States of America

**Carolina Vieira**

Brazil

**John Vince**

Papua New Guinea

**Fernando Volpe**

Brazil

**Jennifer Wagman**

United States of America

**Bobby Webster**

United States of America

**Mary Nell Wegner**

United States of America

**Maxine Whittaker**

Australia

**Mandika Wijeyaratne**

Sri Lanka

**Erin Wolff**

United States of America

**Kapil Yadav**

India

**Hakan Yarali**

Turkey

**Engida Yisma**

Ethiopia

**Mezgebu Yitayal**

Ethiopia

**Jun Yoshinaga**

Japan

**Shehla Zaidi**

Pakistan

**Sanjay Zodpey**

India

